# The frequency of cancer predisposition gene mutations in hereditary breast and ovarian cancer patients in Taiwan: From *BRCA1/2* to multi-gene panels

**DOI:** 10.1371/journal.pone.0185615

**Published:** 2017-09-29

**Authors:** Pi-Lin Sung, Kuo-Chang Wen, Yi-Jen Chen, Ta-Chung Chao, Yi-Fang Tsai, Ling-Ming Tseng, Jian-Tai Timothy Qiu, Kuan-Chong Chao, Hua-Hsi Wu, Chi-Mu Chuang, Peng-Hui Wang, Chi-Ying F. Huang

**Affiliations:** 1 Institute of Clinical Medicine, National Yang-Ming University, Taipei, Taiwan; 2 Department of Obstetrics and Gynecology, Taipei Veterans General Hospital, Taipei, Taiwan; 3 Department of Obstetrics and Gynecology, National Yang-Ming University, Taipei, Taiwan; 4 Department of Oncology, Taipei Veterans General Hospital, Taipei, Taiwan; 5 Department of Surgery, Taipei Veterans General Hospital, Taipei, Taiwan; 6 Department of Biomedical Sciences, Chang Gung University, New Taipei City, Taiwan; 7 Department of Obstetrics and Gynecology, Chang Gung Memorial Hospital, Taoyuan, Taiwan; 8 Department of Medical Research, China Medical University Hospital, Taichung, Taiwan; 9 Institute of Biopharmaceutical Sciences, National Yang-Ming University, Taipei, Taiwan; Ohio State University Wexner Medical Center, UNITED STATES

## Abstract

An important role of genetic factors in the development of breast cancer (BC) or ovarian cancer (OC) in Taiwanese (ethnic Chinese) patients has been suggested. However, other than germline *BRCA1* or *BRCA2* mutations, which are related to hereditary breast-ovarian cancer (HBOC), cancer-predisposition genes have not been well studied in this population. The aim of the present study was to more accurately summarize the prevalence of genetic mutations in HBOC patients using various gene panels ranging in size from *BRCA1/2* alone to multi-gene panels. Among 272 HBOC patients analyzed, the prevalence of *BRCA1*, *BRCA2* and *non-BRCA1/2* pathogenic mutations was 7.7% (21/272), 6.8% (16/236) and 8.2% (13/159), respectively. The total mutation rate was 18.4% (50/272). Although no founder mutations were identified in this study, two recurrent mutations, *BRCA1* (c.3607C>T) and *BRCA2* (c.5164_5165 delAG), were found. The main pathogenic/likely pathogenic mutations in non-*BRCA1/2* genes included *ATM*, *BRIP1*, *FANCI*, *MSH2*, *MUYTH*, *RAD50*, *RAD51C and TP53*. The prevalence rate of gene mutations in HBOC patients did not differ with respect to whether BC or OC was the first diagnosis or they presented a family history of the disease or their age at diagnosis. HBOC patients with both BC and OC exhibited a higher prevalence rate of mutations (50.0%) than patients with OC (25.0%) or BC (8.6%) alone. In conclusion, evaluation of hereditary cancer risk in Taiwan HBOC patients, particularly individuals with double cancer, is strongly encouraged. Panel testing can yield additional genomic information, and widespread and well-designed panel testing will help in assessing more accurate mutational prevalence of risk genes.

## Introduction

Ovarian cancer (OC) continues to be the leading cause of death from gynecological cancer [1]. Breast cancer (BC) is the most prevalent malignancy in women in western countries [2].In Taiwan, BC is the leading cause of death in women, and OC has the highest mortality among all gynecological cancers [3]. Although the majority of BC and OC cases are sporadic, approximately 10% of ovarian cancer cases and 3–5% of BC cases are due to germline mutations in the genes *BRCA1* and *BRCA2* [4–6], which has been described as hereditary breast-ovarian cancer (HBOC) syndrome [5,7]. Individuals who carry mutations in either of these genes have a 47% to 55% probability of developing BC and a 17% to 39% risk of developing OC by the age of 70 [8,9].

HBOC is characterized by a young age of onset, multiple primaries, bilateral BC, and a history of first- or second-degree family members with similar diagnoses [4,6,10,11]. These patients are typically referred to medical genetics specialists by surgical oncologists, oncologists, gynecologists and gynecologic oncologists. There is a well-established counseling strategy based on comprehensive mutation-based management guidelines for carriers or families with *BRCA1*/*2* mutations, and patients benefit from early intervention or prevention of cancer. Taiwan’s National Health Insurance (NHI) is known worldwide for its low-cost, convenient, and rapid disease examination as well as treatment and follow-up procedures. However, genetic counseling and testing for HBOC patients are not common or easily accessible for three reasons. First, there are few official, licensed medical genetic specialists and physicians with training in cancer genetics. Second, prior to the *“Angelina* effect”, patients diagnosed by a gynecologist or surgical oncologist were typically unaware of the importance of genetic testing, and doctors have little time to conduct pre-test screening due to the short out-patient time for each patient. Third, Taiwan’s NHI does not cover the fee for *BRCA1/2* testing and subsequent preventative surgery, even when positive results are obtained. Altogether, these circumstances result in a limited number of patients seeking *BRCA1/2* testing, and large studies are seldom reported. Therefore, the frequency of mutations reported in Taiwan varies significantly in previous studies, ranging from 1.6% to 8.5% [12–15]. Moreover, target populations have largely involved BC patients identified by a surgical oncologist or OC patients identified by a gynecologist, whereas additional information from the entire spectrum of HBOC patients in Taiwan remains unavailable though is urgently needed for first-line doctors.

Mutational analysis of *BRCA1*/*BRCA2* is laborious due to the large sizes of these genes as well as the diversity of mutations. Traditional techniques include direct sequencing, denaturing gradient gel electrophoresis (DGGE), and denaturing high-performance liquid chromatography (dHPLC). Direct sequencing is expensive and time-consuming; the other two methods are complex, and results often require re-evaluation by direct sequencing [16]. Thus far, some cancer-predisposition genes, such as *TP53* (Li-Fraumeni syndrome), *PTEN* (Cowden syndrome), *STK11* (Peutz-Jeghers syndrome), and *CDH1* (hereditary diffuse gastric cancer syndrome/hereditary diffuse gastric cancer syndrome) have been associated with a moderate-to-high risk of BC or OC [16–20]. Re-sequencing microarrays and next-generation sequencing (NGS) [21] enable inexpensive, rapid multi-gene testing for clinical applications. Indeed, the reduced cost of these techniques has resulted in the widespread application of multi-gene panels, with greater benefits than limited *BRCA1/2* testing [22–26]. Furthermore, certain mutations or potentially pathogenic mutations may alter medical care. For example, according to Kurian et al. [27], multiple-gene sequencing identified 16 potentially pathogenic mutations, allowing for early detection of a precancerous colon polyp. Tedaldi et al. [28] reported that it is difficult to distinguish the clinical features and age at diagnosis of patients with *BRCA1/2* mutations from those with non-*BRCA1/2* gene mutations. Although Lin et al. reported the mutational profile of 133 Taiwanese patients with early-onset, bilateral, familial BC using a 68-gene panel [15], the potential benefits of switching from two-gene to multi-gene panels for HBOC patients, including OC patients and healthy individuals with a family history, in Taiwan are unknown.

In the present study, we aimed to accurately analyze the prevalence of mutations in *BRCA1*, *BRCA2* and other cancer-predisposition genes in 272 Taiwanese patients with suspected HBOC based on both a cross-sectional hospital cohort and meta-analysis of published reports. Furthermore, we sought to provide additional pre-test information for first-line surgical oncologists and gynecologists to use when determining whether to refer or offer a genetic test to their HBOC patients.

## Materials and methods

### Study population

#### Hospital cohort

**Fig 1:**
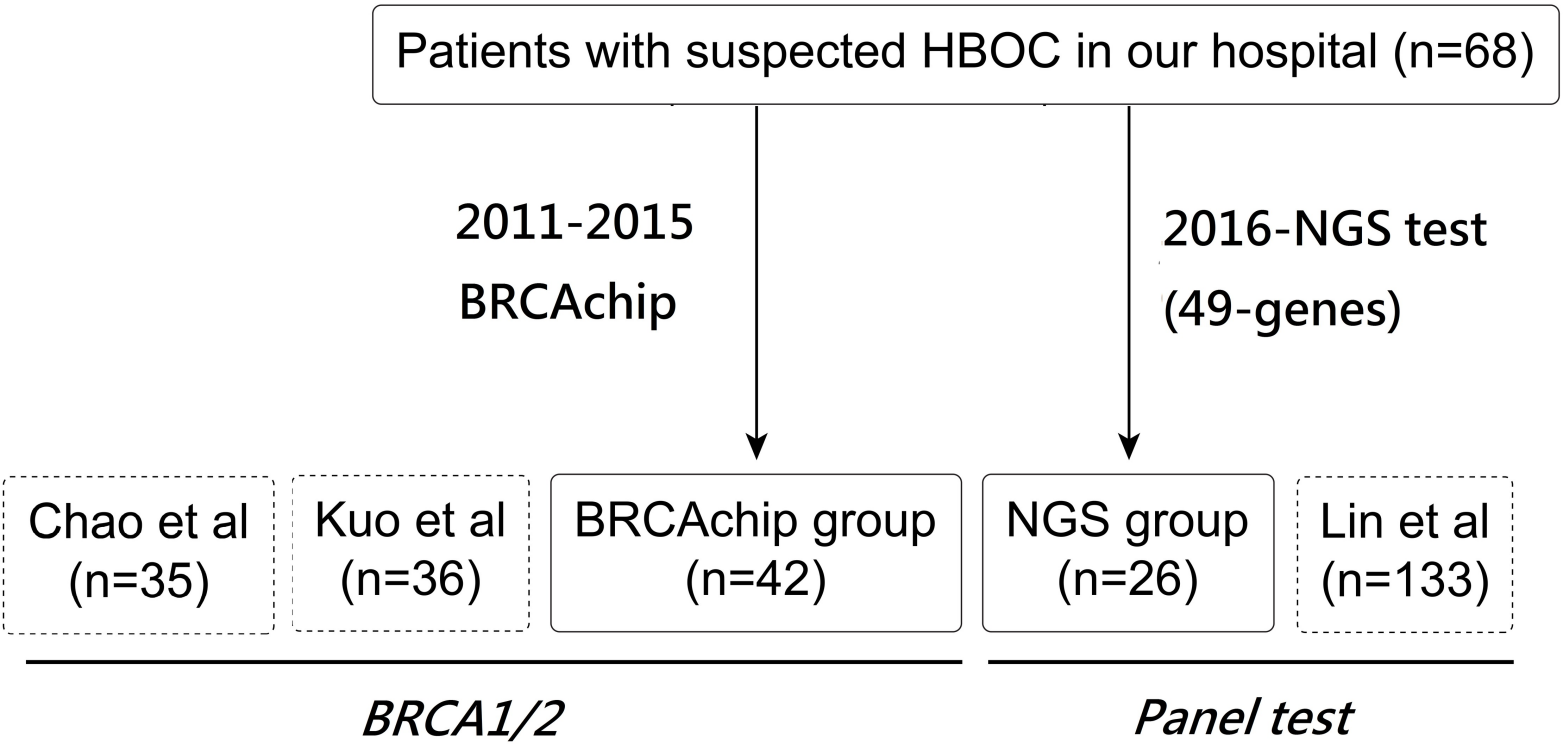
A flowchart of our study design. This flowchart illustrates the cross-sectional hospital cohort and meta-analysis which were divided into two groups: *BRCA1/2* and *Panel test*.

A cross-sectional hospital cohort of 68 women with HBOC who were referred for genetic testing between January 2011 and December 2016 at Taipei Veterans General Hospital was examined in the present study. All patients met at least one of the following HBOC criteria: (1) early-onset BC (at 50 years of age or younger); (2) early-onset BC and at least one first- or second-degree relative with BC or OC/tubal cancer/peritoneal cancer; or breast cancer at any age with two or more close relatives with breast cancer at any age (3) a personal history of both BC and OC; (4) OC/tubal cancer/peritoneal cancer at any age; (5) co-occurrence of BC or OC /tubal cancer/peritoneal cancer with another type of cancer in the same person, male BC or bilateral, triple-negative or estrogen receptor (+) BC at any age; (6) at-risk healthy patients with a family history [6,10,11,29]. Pedigrees, clinical information such as gender, age of diagnosis, tumor histological type and clinical stage, and the cancer history of family members were obtained from all patients. Among the 68 patients, 42 received a BRCAchip test for only *BRCA1/2* before January 2016, and 26 received testing using a 49-gene panel via the NGS method after January 2016 (Fig 1). Eighteen patients with BRCAchip provided consent for their medical records to be used in this study. The specimens and clinical data were collected under the protocol approved by the institutional review board of Taipei Veterans General Hospital (2011-08-017GB; Hereditary breast and ovarian cancer syndrome: detection of BRCA1/2 and TP53 gene mutation). The clinical data of the other 50 patients, including 24 BRCAchip and 26 panel test patients were analyzed anonymously and reported.”.

### Mutation screening

#### *BRCA1*/*2*: Re-sequencing array (BRCAchip)

Prior to January 2016, the re-sequencing array BRCAchip was designed by Vita Genomics, Inc. (Taipei, Taiwan), and Multiplex Ligation-Dependent Probe Amplification methods were used for *BRCA1*/*2* testing. The array was manufactured by Affymetrix (Santa Clara, CA, USA). The technique has been previously described by Liu et al. [30]. To expand the mutation detection function, the BRCAchip array was divided into two sections in the following manner: main tiling, primarily for the detection of single-nucleotide variants; and extra tiling, to detect 456 specific small insertions or deletions (indels) in *BRCA1* and *BRCA2* (249 indels for *BRCA1* and 207 indels for *BRCA2*), which were selected from Breast Cancer Information Core Database (BIC database, NHGRI). The BRCAchip arrays were scanned using an Affymetrix GeneChip scanner 3000 7G to create CEL files for subsequent analysis. Analysis of the BRCAchip data was performed using Affymetrix GeneChip® Sequence Analysis Software (GSEQ) 4.0, employing the ABACUS (Adaptive Background Genotype Calling Scheme) algorithm with optimized settings. Briefly, base calls were directly deposited into a database with a user interface in the “VitaMINE” computer system (Vita Genomics Inc., Taipei, Taiwan), which provided all of the nucleotide differences in the called sequences compared with the reference sequence obtained from GenBank. The nucleotide differences and bases that could not be called using the algorithm were re-evaluated by manual assessment of signal intensity plots. To verify the performance of BRCAchip, complete dideoxy sequencing of all exons was performed for subjects from an in-house study, with a chip call accuracy of 99.99% (426,206/426,207) (data not shown). In the present study, DNA sequencing of patients with mutations was also performed using the fluorescently labeled dideoxy chain termination method with Big Dye Terminator ABI Prism Kit and an ABI PRISM^TM^ 3730 DNA Analyzer (Applied Biosystems, Foster City, CA, USA), according to the manufacturer’s instructions.

#### Multi-gene panel test: Library construction, target region capture, and massively parallel sequencing

The 49-gene NGS panel has been described previously by Guan et al. [17]. One microgram of DNA from each sample was used for library construction. Briefly, genomic DNA fragmentation was performed to generate fragments with a peak of 250 bp, followed by purification using AMPure beads (Beckman Coulter, Brea, USA), with three subsequent enzymatic steps, end repair, A-tailing, and ligation to Illumina adapters, according to the standard library construction protocol. The libraries were quantified using a Bioanalyzer 2100 instrument (Agilent Technologies, Palo Alto, USA). DNA target enrichment was performed on a custom sequence capture-array (Roche, Basel, Switzerland). The pooled library was used for target-region capture hybridization. The size and quantity of the captured library was assessed using a Bioanalyzer 2100 instrument, and enrichment of the target region was assayed via quantitative polymerase chain reaction (qPCR). Sequencing was then performed with 2 x100 bp paired-end reads and 8-bp index reads using a HiSeq2500 Analyzer (Illumina, San Diego, USA), with standard cluster generation and sequencing according to the manufacturer’s instructions. To accurately monitor the experimental sample, we selected 21 single-nucleotide polymorphisms (SNPs) to distinguish each sample; all 21 SNPs in each sample were evaluated via matrix-assisted laser desorption/ionization-time-of-flight mass spectrometry (MALDI-TOF MS); for each sample, the NGS results were confirmed by the MS results.

#### Mutational analysis and web-server prediction programs (*in silico* bioinformatic analysis)

Online databases, including the Human Gene Mutation Database, Single Nucleotide Polymorphism Database (dbSNP), 1000 Genomes, HapMap, and the BIC (Breast Cancer Information Core, http://research.nhgri.nih.gov/bic/) database, as well as other online search engines (ClinVar (http://www.ncbi.nlm.nih.gov/clinvar/), LOVD (Leiden Open Variation Database, http://www.lovd.nl/3.0/ home), ARUP (http://arup.utah.edu/database/BRCA/), BRCA Share (http://www.umd.be/BRCA1/)[31], and BRCA Exchange (http://brcaexchange.org/)) were used to search for variant classifications. We followed the American College of Medical Genetics and Genomics (ACMG) 2015 guidelines for interpretation of germline variants [32]. Briefly, reported variants that produced premature termination codons associated with non-functional or truncated proteins (very strong evidence of pathogenicity; PVS1) were classified as pathogenic (P), including nonsense mutations, frameshift mutations, splice-site mutations and exonic deletions. Novel mutations without well-established functional studies were classified as likely pathogenic (LP). According to the classification, if a variant does not fulfill criteria for pathogenic/likely pathogenic or benign/likely benign or the evidence for benign and pathogenic is conflicting, the variant is classified as "uncertain significance". Two web-servers for bioinformatic missense variant prediction, Poly-Phen-2 (Polymorphism Phenotyping, v2) [33] and Sorting Intolerant from Tolerant (SIFT) [34], were used to evaluate the pathogenicity of missense mutations. PolyPhen-2 (http://genetics.bwh.harvard.edu/pph2/) predicts the pathogenicity of an amino acid substitution based on structural alterations. SIFT (http://sift.jcvi.org/) is a sequence homology-based tool for the identification of well-conserved positions between protein species that are typically predicted as deleterious.

### Literature search strategy and selection criteria

To conduct a meta-analysis of prevalence, a comprehensive literature search of PubMed was performed in December 2016. This search was limited to English-language publications and articles published since January 2010. For studies related to the probability of identifying a germline *BRCA1/2* mutation and other gene mutations conducted in Taiwan, the following search strategy was employed: ((((probability) OR frequency)) AND (((BRCA) OR *BRCA1*) OR *BRCA2* OR NGS) AND ((((ovarian cancer) OR ‘fallopian tube cancer’) OR ‘peritoneal cancer’) OR ‘Breast cancer’) AND (Taiwan). Five articles were identified [12–14,35]. Reports by Chen et al. [36] and Wang et al. [13], which only involved screening of the *BRCA1* gene without the *BRCA2* in HBOC populations, were discarded. Three articles, published by Kuo et al. (n = 36) [14], Lin et al. (n = 133) [15] and Chao et al. (n = 99) [12], were selected for comprehensive analysis of *BRCA1/2*. The study by Lin et al. [15] included other non-*BRCA1/2* genes (Table 1). Combined with our cohort, 272 HBOC patients were included in the present study, which were divided into two methodological groups (*BRCA1/2* and *Panel test*) (Fig 1).

**Table 1:** Frequency of germline pathogenic mutations in Taiwanese HBOC patients.

Reference	Population	No. of patients	No. of mutation cases (%)				Methods
			*BRCA1*	*BRCA2*	Non-BRCA	Total	
Kuo et al.[14]	Early-onset, bilateral or familial BC	36	3 (8.3)	N/A	N/A	3 (8.0)	BRCAChip (re-sequencing microarray)
Chao et al. [12]	Ovarian cancer	35	3 (8.6)	2 (5.7)	N/A	5 (14.2)	NGS (*BRCA1/2*) for FFPE*^¶^
Lin et al. [15]	Early-onset, bilateral or familial BC	133	9 (6.7)	11 (8.2)	10 (7.5)	30 (22.5)	NGS (68-gene panel)^¶^
Present study	At-risk patients with HBOC	42	5 (11.9)	2 (4.8)	NA	7 (16.7)	BRCAChip (re-sequencing microarray)
		26	1 (3.8)	1 (3.8)	3 (11.5)	5 (19.2)	NGS (49-gene panel)^¶^
Total		272	21 (7.7)^a^	16 (6.8)^b^	13 (8.2)^c^	50(18.4)^a^	

HBOC: hereditary breast and ovarian cancer; NGS: next-generation sequencing

*FFPE: formalin-fixed paraffin-embedded normal tissue used to obtain germline information.

^a^Included Kuo[14], Chao [12], Lin [15] and the present study (total n = 272).

^b^Included Chao [12], Lin [15] and the present study (total n = 236).

^c^Included Lin [15] and NGS of the present study (total n = 159).

^¶^ Detailed list of genes and detected pathogenic genes are provided in S2 Table

## Results

### Distribution of variations in *BRCA1/2* and other non-*BRCA* genes

**Fig 2:**
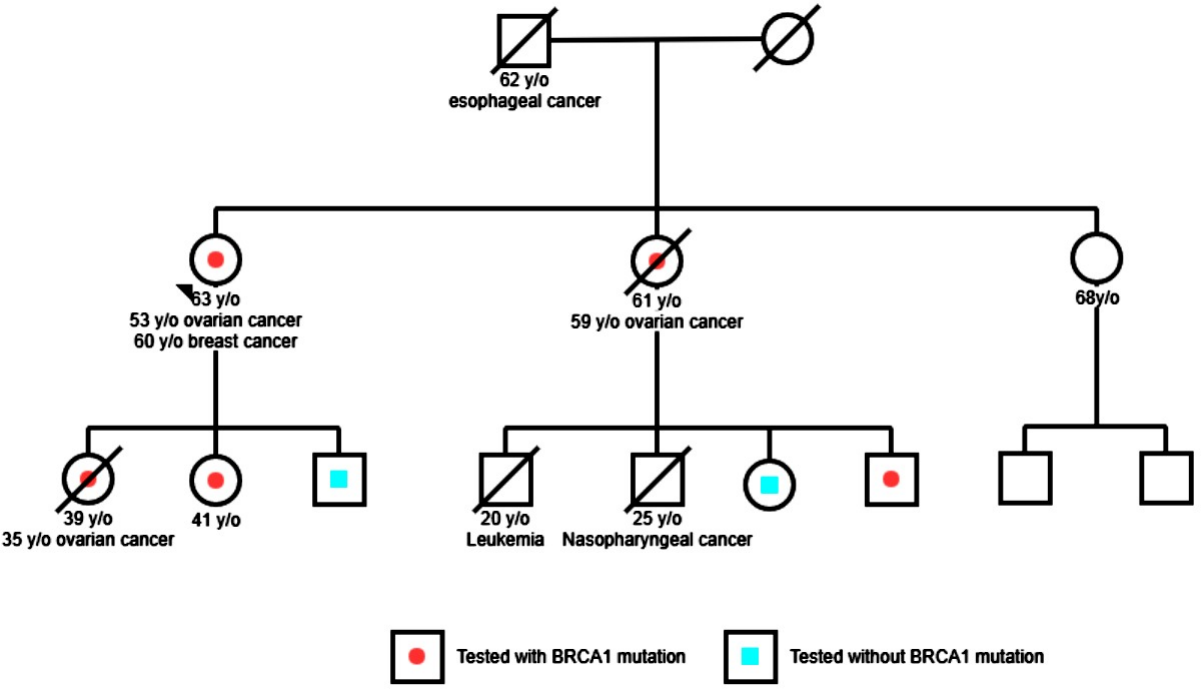
Family #42 pedigree. The index individual #42 in this family has BRCA1 p.V802*. mutation. Individuals with ovarian, breast or other cancer with age at diagnosis are mentioned. Individuals who received test with positive BRCA1 mutation are marked with ah a red dot. Individuals who received test without mutation are marked with a blue square.

The mean age of the 68 patients was 44.4±11.7 years-old (range 22–74). The characteristics of two groups are in S1 Table. Eleven pathogenic/likely pathogenic variants (11/68; 16.2%) were found among the 68 patients, including 5 *BRCA1* variants in 6 patients (6/68; 8.8%), 3 *BRCA2* variants in 3 patients (3/68; 4.4%), 2 *RAD50* variants in 2 patients (2/68; 2.9%) and 1 *BRIP1* variant in 1 patient (1/68; 1.4%) (Table 2). The sequence variations comprised 5 nonsense mutations (5/11; 45.5%), 4 frame-shift mutations (4/11; 36.4%) and 1 splice-site mutation (1/11; 9%). Of them, 1 *BRCA1* genetic variant (c.3472delG), 1 *BRCA2* mutation (c.1036delAA in exon 10), 1 *RAD50* mutation (c.1717delA) and 1 *BRIP1* mutation (c.2244C>G) were identified as likely pathogenic mutations not reported in any database or previous study (Table 2). The family pedigree #42 is demonstrated in Fig 2.

**Table 2:** Clinical characteristics of patients with pathogenic/likely pathogenic variants in a hospital cohort.

Gene	Case No.	HGVS cDNA	AA change	Cancer (Age)	Family history (age)	Reported/novel	Clinical relevance
***BRCA1***	#7 #9	c.3607C>T	p.R1203*	#7 BC (40) Tubal ca (67) #9 OC(43)	#7 grandfather: laryngeal ca #9 no family history	Reported (ClinVar, BX,BS,AURP),	Pathogenic
	#15	c.3637G>T	p.E1213*	BC (39) Ureter ca (60)	No family history	Reported (JCO)[37]	Pathogenic^$^
	#22	c.5332+1G>A		OC (42) BC(62)	No family history	Reported (ClinVar, BX, AURP)	Pathogenic
	#42	c.2393_2393delC (2512delC)	p.V802*	OC (53) and BC (60)	Sister: OC (59), daughter: OC (35), father: esophageal ca (62)	Reported (ClinVar,BX)	Pathogenic
	#57¶	c.3472delG	p.E1158Kfs*2	OC(55)	No family history	*Novel*	Likely pathogenic^$^
***BRCA2***	#34	c.1036delAA	p.N346fs_S356*	BC (26)	Maternal grandmother (40) and maternal aunt (70): BC	*Novel*	Likely pathogenic^$^
	#18	c.7977-1G>T	IVS17-1G>T	BC (68)	Father (82), sister (36) and daughter (unknown): BC	Reported (ClinVar,BX)	Pathogenic(ClinVar/not reviewed(BX)
	#63¶	c.7567_7568delCT	p.L2523EfsTer15	BC (58) OC (61)	No family history	Reported (ClinVar,BX)	Pathogenic
***RAD50***	#57¶	c.1717delA	p.K574Nfs*24	OC (55)	No family history	*Novel*	Likely Pathogenic^$^
	#50¶	c.3553C>T	p.R1185*	BC (33)	Aunt (unknown): BC	Reported (ClinVar)	Pathogenic
***BRIP1***	#60¶	c.2244C>G	p.Y748*	PPSC (56)	No family history	*Novel*	Likely Pathogenic^$^

Abbreviations: OC: ovarian cancer; BC: breast cancer; tubal ca: fallopian tube cancer; PPSC: primary peritoneal serous carcinoma. ClinVar: https://www.ncbi.nlm.nih.gov/clinvar/; BX (BRCAexchange): http://brcaexchange.org/; BS(BRCAshare): http://www.umd.be/BRCA1/ AURP: http://arup.utah.edu/database/BRCA/

JCO: *BRCA1* and *BRCA2* mutation frequency in women evaluated in a breast cancer risk evaluation clinic [37]

¶detected by the NGS panel.

^$^fulfilled 2015 ACMG criteria for likely pathogenic variant: 1PVS1 ((null variant framshift change or nonsense mutation) + 1 PM2 (absent from control)[32]

A total of 13 missense variants of uncertain significance (VUS) mutations were identified in *RCA1*, *BRCA2*, *TP53*, *PALB2*, *MUTYH*, *RAD50*, *CHEK2*, and *CDH1* (Table 3). The pathogenicity of these missense mutations was predicted using two *in silico* programs (SIFT and polyphen-2), and the results are provided in Table 3.

**Table 3:** Clinical characteristics and bioinformatic analysis of variants of uncertain significance in the hospital cohort.

Gene	Case No.	HGVS cDNA	AA change	Cancer (Age)	Family history (age)	Reported/novel	Clinical relevance	Bioinformatic analysis	
								Polyphen-2	SIFT
***BRCA1***	#28	c.571G>A	p.V191I	BC (57)	Mother(unknown age):cervical cancer	Reported (ClinVar; BX,BS, AURP)	Benign	Benign	Damaging
	#32	c.2286A>T	p.R762S	BC (27)	No family history	Reported (ClinVar, BX) (	Uncertain Significance**	Benign	Damaging
***BRCA2***	#7	c.440A>G	p.Q147R	BC (40) Tubal ca (67)	No family history	Reported (ClinVar; BX,)	Uncertain Significance**	Benign	Tolerated
	#15	c.10075G>A	p.E3359K	BC (39) Ureter ca (60)	No family history	Reported (dbSNP, BX)	Uncertain Significance**	Benign	Damaging
	#13 #34	c.6322C>T	p.R2108C	#13 OC (39) #34 BC (26)	#13 no family history #34 maternal grandmother (40) and maternal aunt (70): BC	Reported (ClinVar; BX,)	Likely benign**	Benign	Tolerated
***TP53***	#38^¶^	c.532 C>G	p.H178D	BC (28)	Maternal aunt (65): BC	Reported (IARC) ^&^	Uncertain Significance**	Probably damaging	Deleterious^&^
***PALB2***	#26^¶^	c.3054G>C	p.E1018D	BC (49)	Mother (75) and sister (46): BC	Reported (ClinVar)	Uncertain Significance**	Probably damaging	Damaging
**MUTYH**	#44^¶^	c.715G>A	p.V225I	BC (30)	Mother (51): BC	Reported (ClinVar)	Uncertain Significance**	N/A	Damaging
**RAD50**	#49^¶^	c.323A>G	p.K108R	No cancer (24)	Mother (30): BC	Reported (ClinVar)	Uncertain Significance**	Probably damaging	Tolerated
**CHEK2**	#51^¶^	c.1111C>T	p.H371Y	BC (66)	Sister: uterine cancer (50) Niece: OC (20)	Reported (ClinVar)	Uncertain Significance**	Benign	Damaging
**CDH1**	#52^¶^	c.2474C>T	p.P825L	Cervical adenosarcoma (43)	Sister (30): BC	Reported (ClinVar)	Uncertain Significance**	Probably damaging	Damaging
**MLH1**	#54^¶^	c.2174G>A	p.R725H	BC (67)	Mother (unknown age): BC Father(unknown age): lung cancer	Reported (ClinVar)	Uncertain Significance**	Probably damaging	Damaging

Abbreviations: OC: ovarian cancer; BC: breast cancer; tubal ca: fallopian tube cancer; ClinVar: https://www.ncbi.nlm.nih.gov/clinvar/; BX (BRCAexchange): http://brcaexchange.org/; BS (BRCAshare): http://www.umd.be/BRCA1/;AURP: http://arup.utah.edu/database/BRCA/

^&^ reported in the IARC TP53 database (http://p53.iarc.fr/)

^¶^detected in the NGS panel.

**These variations did not fulfill 2015 ACMG guidelines as “pathogenic or likely pathogenic” [32] but were classified as “uncertain significance” by some labs in the ClinVar database.

### Prevalence of pathogenic *BRCA1/2* and non-*BRCA* mutations in HBOC patients

Based on a review of selected published research, the target populations of Kuo et al. (n = 36) [14] and Lin et al. (n = 133) [15] comprised women with early-onset, bilateral and familial BC. Kuo et al. [14] only provided *BRCA1* results, whereas *BRCA2* results were not available. By using formalin-fixed paraffin-embedded normal tissue to obtain germline information, Chao et al. examined 99 patients but only reported 35 germline results, using formalin-fixed paraffin-embedded normal tissue to obtain germline information [12]. Combined with our cohort, 272 HBOC patients were included in the present study, which were divided into two methodological groups (*BRCA1/2* and *Panel test*) (Fig 1). The prevalence of *BRCA1*, *BRCA2* and non*-BRCA1/2* pathogenic mutations was 7.7% (21/272), 6.8% (16/236) and 8.2% (13/159), respectively. The total mutation rate was 18.4% (50/272) (Table 1). The non-*BRCA1/2* genes detected in the present study included *ATM*, *BRIP1*, *FANCI*, *MSH2*, *MUYTH*, *RAD50*, *RAD51C and TP53*. (S2 Table). One recurrent *BRCA1* (c.3607C>T) mutation was detected in one BC and tubal cancer patient and in one OC patient. Another recurrent *BRCA2* (c.5164_5165 delAG) mutation, previously observed by Chao et al. [12], was detected in two OC patients.

### Mutation rate in populations with different family histories, ages at diagnosis, and personal histories

Regarding common HBOC criteria, such as family history, age at diagnosis, and personal history, we found that the prevalence rate of gene mutations in HBOC patients did not differ with respect to whether patients were diagnosed with BC as the first diagnosis, whether they presented a family history of the disease, or their age at diagnosis (18.8% *vs* 12.8%, *vs 16*.*7%; p* = 0.68), with the exception of late-onset patients without a family history (no patients) (Table 4). Most OC patients were diagnosed at more than 50 years of age. The frequencies between patients diagnosed with OC as the first diagnosis were similar (50.0% *vs* 26.4%*vs* 55.5%, *p* = 0.69), but the OC group did not include patients with a family history plus early-onset disease (Table 4). Regarding personal history, HBOC patients with both BC and OC exhibited a higher prevalence rate of mutations (50.0%) than patients with OC (25.0%) or BC (8.3%) alone (*p* = 0.03) (Table 5).

**Table 4:** Distribution of pathogenic/likely pathogenic mutations with respect to family history, age at diagnosis and first cancer at diagnosis.

Family history (FH)^¶^ and age at diagnosis	Breast cancer as first diagnosis^a^						Ovarian cancer as first diagnosis^b^					
	*BRCA11/2*% (positive/total cases)		*Panel test* % (positive/total cases)			Total % (positive/total cases)	*BRCA1/2*% (positive/total cases)		*Panel test* % (positive/total cases)			Total % (positive/total cases)
	*BRCA1*	*BRCA2*	*BRCA1*	*BRCA2*	*Non-* *BRCA*		*BRCA1*	*BRCA2*	*BRCA1*	*BRCA2*	*Non-* *BRCA*	
FH(+) and Early onset^&^	0	9	5.8	10.1	4.3	18.8	0	0	0	0	0	0
	(0/11)	(1/11)	(4/69)	(7/69)	(3/69)	(15/80)	(0/0)	(0/0)	(0/1)	(0/1)	(0/1)	(0/1)
FH(+) Late onset^&^	0	33.3	2.2	4.5	4.5	12.8	50	0	0	0	0	50.0
	(0/3)	(1/3)	(1/44)	(2/44)	(2/44)	(6/47)	(1/2)	(0/2)	(0/0)	(0/0)	(0/0)	(1/2)
FH(-) and Early onset^&^	25	0	2.9	5.8	2.9	16.7	25	0	0	0	0	26.4
	(2/8)	(0/8)	(1/34)	(2/34)	(2/34)	(7/42)	(4/10)	(0/10)	(0/1)	(0/1)	(0/1)	(4/11)
FH(-) and Late onset^&^	0	0	0	0	0	0	0	60	25	0	25	55.5
	(0/5)	(0/5)	(0/0)	(0/0)	(0/0)	(0/5)	(0/5)	(3/5)	(1/4)	(0/4)	(1/4)	(5/9)
						*P*^*c*^ = 0.68						*P*^*d*^ = 0.69

^¶^At least one first- or second-degree family member with HBOC cancer

^&^Early onset, < 50 years of age; late onset ≥ 50 years of age.

^a^Included Lin [15] and the present study (total n = 174).

^b^Included 5 cases with available FH of Chao [12] the present study (total n = 23).

^c^. Three groups (FH(+) and early onset; FH(+) and late onset; FH(-) and early onset): calculated using Chi-square and Fisher's exact tests.

^d.^ Three groups (FH(+) and late onset; FH(-) and early onset; FH(-) and late onset); calculated using Chi-square and Fisher's exact tests.

**Table 5:** Distribution of pathogenic/likely pathogenic mutations with respect to personal history.

Personal history	*BRCA1*	*BRCA2*	*Non-BRCA*	Total (%)
Ovarian cancer (n = 16)	3	0	1	4 (25.0)
Breast cancer (n = 36)	0	2	1	3 (8.3)
Two cancers (n = 6)^¶^	2	1	0	3 (50.0)
			Ovarian: BRIP1 (1)	*p* = 0.03*
			Breast: RAD50 (1)	

^¶^6 patients with BC and OC

*Statistically significant, calculated using the Chi-square test.

## Discussion

Based on a single hospital cohort and meta-analysis, this studies recruited the largest Taiwanese HBOC population (n = 272). The prevalence of *BRCA1* and *BRCA2* mutations was found to be 7.7% (21/272) and 6.8% (16/236), respectively (Table 1). The ethnic profile of Taiwan is 95% Han Chinese, and most of our ancestors had migrated from Mainland China [38]. A large study of Mainland Chinese familial BC and OC patients revealed an estimated prevalence of 10.6% for *BRCA1* and 5.2% for *BRCA2* [39]. Regarding other Asian ethnic HBOC populations, the prevalence rates of clearly deleterious *BRCA1* and *BRCA2* mutations were reported to be 6.7% (6 of 90) and 8.9% (8 of 90), respectively, in a study from Singapore published by Ang et al. [40]. The *BRCA1/BRCA2* mutation rates observed in the present study were similar (7.7% *vs* 6.7–10.6%; 6.8% *vs* 8.9–5.2%) to those reported in other Asian studies but lower than those reported for Europe and North America [41]

The prevalence rate of non-*BRCA1/2* predisposing genes detected using NGS methods is gradually being analyzed in Europe and North America, though little is known about the prevalence rate in Asian populations. LaDuca et al. [42] have examined multi-gene panels for hereditary cancer-predisposition testing and reported positive rates of 7.4% for BreastNext and 7.2% for OvaNext. Additionally, a rate of 4.6% was reported for other BC/ovarian cancer predisposing genes in a BC population in the US [42]. Castéra et al. reported a rate of 4.8% (34/708) in a French HBOC population using different versions of the NGS capture set [25]. We observed that the prevalence of non-*BRCA1*/2 genes was 7.5% based on 68 genes, 11.5% based on 49 genes and 8.2% based on all cases in our study (Table 1). By using a 30-gene panel, a study of South Indian women with HBOC found a mutation rate of 9.8% among non-*BRCA1/2* genes (9/91) [43]. Moreover, Wong et al. identified 47.8% of pathogenic variants in non-*BRCA1/2* genes among 220 HBOC patients in Singapore [44], and a mutation rate of 6% was observed for non-*BRCA1/2* genes based on a 15-gene panel in a Malaysian BC patient study [45]. Therefore, the detection rate of non-*BRCA1/2* genes depends on the platforms and target populations involved.

Some populations, such as those of eastern European (Ashkenazi) Jewish ancestry, exhibit a high frequency (1 in 40). Three mutations (185delAG and 5382insC in *BRCA1* and 6174delT in *BRCA2*) have been defined as founder mutations [46], and three founder mutations, *BRCA1* 5382insC, C61G and 4153delA, are common in Polish familial BC patients [47]. In the Han Chinese population, four recurrent *BRCA1* mutations (c.470_471delCT, c.3342_3345delAGAA,c.5406+1_5406+3delGTA and c.981_982delAT) were found to account for 34.5% (10/29) of *BRCA1* mutations, and four recurrent *BRCA2* mutations (c.2808_2811delACAA, c.3109C.T, c.7436_7805del370 and c.9097_9098insA) were found to account for 40% (16/40) of *BRCA2* mutations in a Hong Kong HBOC population consisting of 451 patients [48]. The most common pathogenic *BRCA1* variant detected in an analysis of a large mainland Chinese population was c.981_982delAT (p.Cys328*), which shows a frequency that is substantially higher in Mainland Chinese populations than in non-Chinese populations (4.4% *vs* 0.1%) in the BIC database [39]. In the same study, the most common pathogenic *BRCA2* variants were found to be c.3195_3198delTAAT (p.Asn1066Leufs*10) (n = 5) and c.5576_5579delTTAA (p.Ile1859Lysfs*3) (n = 5) in exon 11 [39]. The *BRCA2* mutation c.7480C>T is enriched in Korean familial BC patients [49], and Wong et al. reported four recurrent mutations in *BRCA1* (p.Y1127* (c.3381T4A), E23Rfs*18 (c.67_68delinsAG), p.E1112Nfs*5 (c.3333delA), p.T1691K (c.5072C4)) and one *BRCA2* mutation (p.C161W (c.483T4G)) in a population from Singapore [50]. In our hospital cohort, we detected one recurrent *BRCA1* mutation (p.R1203X (c.3607C>T)) in one double cancer patient and in one OC patient without a family history (Table 2), and the *BRCA2* p.S1722fs (c.5164_5165 delAG) mutation was detected in two OC patients [12]. These two recurrent mutations were not assessed through haplotype analyses and not observed in previous reports on any Han Chinese or other Asian population. Regarding non-*BRCA1/2* genes, the recurrent mutations *PALB2* p.A38G (c.113C4G), *CHEK2* p.R223C (c.667C4T) and *RAD51D* p.I311N (c.932T4) were observed in a population from Singapore [44], though these mutations were not detected in the present study. In contrast, the non-*BRCA1/2* genes we detected included *ATM*, *BRIP1*, *FANCI*, *MSH2*, *MUYTH*, *RAD50*, *RAD51C and TP53*. *T*hus, additional studies examining recurrent non-*BRCA1*/*2* genes in Asian populations are needed.

Training in cancer genetics and genetic counseling and in the use of specific assessment tools for the evaluation of HBOC patients requires considerable time, and such evaluations are difficult for less-trained surgical oncologists and gynecologists to perform in Taiwan as well as in some other countries. Family history, personal history and age at diagnosis are the main parameters in such assessment tools and represent the primary criteria for HBOC [11,51,52]. In the present study, we observed that family history and age at diagnosis did not affect the detection rate in patients with BC at first diagnosis. The results of the present study are similar to those in a Polish population, whereby 51% of *BRCA1*-positive OC patients and 39% of *BRCA1*-positive BC patients with a negative family history of breast and/or OC among first- and second-degree relatives were identified [53]. We also found that late-onset OC patients, without or with a family history, should receive testing for high prevalence (50.0% and 55.5%; Table 4), which is not the case for BC patients. Recent studies have shown that BRCA testing should be recommend to all women with high-grade serous OC [6]. It is important to prevent OC through the identification of women at an increased risk and the initiation of preventive management, such as bilateral salpingo-oophorectomy prior to developing the disease [51,54]. Another importance issue is that *BRCA1/2* status influences the treatment strategy adopted. Poly (ADP-ribose) polymerase (PARP) is a critical component of base excision repair (BEP) pathway for the repair of single-strand breaks (SSBs). PARP inhibition results in failure of SSB lesion repair but does not affect double-strand break (DSB) repair, which is mainly controlled by both BRCA1 and BRCA2 proteins via homologous recombination [55–57]. Impaired PARP function in BRCA1- or BRCA2-defective cells leads to DNA lesions and cell cycle arrest and/or cell death [58]. Therefore, PARP inhibitors show highly selective synthetic lethality of cells with *BRCA1/2-*dysfunction cancers. In fact, a PARP inhibitor, such as Olaparib [59] and Niraparib [60], increased the median duration of progression-free survival in BRCA-related OC. Conclusively, surgical oncologists and gynecologic oncologist can offer genetic testing to BC or OC patients, regardless of family history or early onset. Considering that *BRCA1*/*2* genes exhibit the highest rate of mutations (50%; Table 5) in patients with breast and OC, patients with double cancers should be intensively aware of genetic testing.

A strength of the present study is that it is the largest summary of Taiwanese HBOC patients; additionally, the results can be provided as part of pre-test counseling. The present study also revealed pathogenic/likely pathogenic genetic variations detected using panels of various sizes, ranging from two-gene to multi-gene panels, in the entire spectrum of Taiwanese HBOC patients. The weaknesses of the present study are associated with its small cohort size and the different NGS platforms involved. Rare mutations, such as large re-arrangements or indels, might not have been observed in the present study. A larger, nation-wide, well-designed survey of HBOC patients using consistent, well-designed NGS panels should be performed. The potential benefits of preventative management for both patients and families after screening may also reduce the financial burden of the NHI program for the treatment of existing cancers.

In conclusion, Taiwan HBOC patients, particularly individuals with double cancer, are strongly encouraged to undergo evaluation of hereditary cancer risk. Panel testing can yield additional genomic information, and widespread and well-designed panel testing will help to obtain more accurate mutational prevalence of risk genes.

## Supporting information

S1 Table Clinical characteristics of both Groups. (DOCX)

S2 Table Detected pathogenic/likely pathogenic genes and their clinical surveillance. (DOCX)
