# Supplementary material for: The frequency of cancer predisposition gene mutations in hereditary breast and ovarian cancer patients in Taiwan: From BRCA1/2 to multi-gene panels
Source: PLoS One. 2017 Sep 29;12(9):e0185615. doi: 10.1371/journal.pone.0185615 (PMC5621677; doi:10.1371/journal.pone.0185615)
Supplement: S1 Table — (DOCX) [file pone.0185615.s001.docx]

| **S1 table. Clinical characteristics of both Groups** | | |
| --- | --- | --- |
|  | BRCAchip group  (n=42) | NGS group  (n=26) |
| Age range (year-old) | 26-67 | 22-74 |
| Mean age (year-old) | 43.1±10.0 | 45.6±14.3 |
| Personal history (%) |  |  |
| Breast cancer | 22 (52.4) | 13 (50.0) |
| Ovarian cancer | 10 (23.8) | 6 (23.1) |
| Two cancer | 7 (16.7) | 1 (3.9) |
| Healthy | 3 (7.1)) | 6 (23.7) |
| HBOC criteria (%) |  |  |
| (1) | 7 (16.7) | 5 (19.2) |
| (2) | 14 (33.3) | 8 (30.1) |
| (3) | 3 (7.1) | 1(3.9) |
| (4) | 12 (28.6) | 6 (23.7) |
| (5) | 3 (7.1) | 0 (0) |
| (6) | 3 (7.1) | 6 (23.7) |
