# Supplementary material for: The frequency of cancer predisposition gene mutations in hereditary breast and ovarian cancer patients in Taiwan: From BRCA1/2 to multi-gene panels
Source: PLoS One. 2017 Sep 29;12(9):e0185615. doi: 10.1371/journal.pone.0185615 (PMC5621677; doi:10.1371/journal.pone.0185615)
Supplement: S2 Table — (DOCX) [file pone.0185615.s002.docx]

| **S2 Table. Detected Pathogenic/Likely Pathogenic Genes and Their Clinical Surveillance** | | | | |
| --- | --- | --- | --- | --- |
| **Panels** | **No.** | **Genes (same genes in red color )** | **Pathogenic/likely pathogenic detected in Taiwan** | **Clinical surveillance** |
| This study | 49 | *APC, ATM, AXIN2, BARD1, BMPR1A, BRCA1, BRCA2, BRIP1, CDC73, CDH1, CDK4, CDKN1B, CDKN2A, CHEK2, EPCAM, EXT1, EXT2, FH, FLCN, MAX, MEN1, MET, MLH1, MLH3, MRE11A, MSH2, MSH6, MUTYH, NBN, NF1, NF2, NTRK1, PALB2, PMS1, PMS2, PTEN, RAD50, RAD51C, RB1, RET, SDHAF2, SDHB, SDHC, SDHD, SMAD4, STK11, TMEM127, TP53, VHL* | *BRCA1(1), BRCA2(1)* | Breast MRI, RRM and RRSO* |
|  |  |  | *BRIP1,(1),* | RRSO* |
|  |  |  | *RAD50(1)* | Monitored for malignancy, particularly breast cancer in women and prostate cancer in men¶. |
| Lin et al. (2016) [15] | 68 | *APC, ARLTS1, ATM, BARD1, BMPR1A, BRCA1, BRCA2, BRIP1, CDH1, CHEK2, DDB1, DDB2, EPCAM, ERCC1, ERCC2, ERCC3, ERCC4, ERCC5, CSB, CSA, FANCA, FANCB, FANCC, FANCD2, FANCE, FANCF, XRCC9, FANCI, PHF9, FANCM, PALB2, SLX4, FGFR2, GT198, ku70, XRCC5, MAP3K1, MDM4, MLH1, MLH3, MRE11, MSH2, MSH3, MSH6, MUTYH, NER, NBS1(NBN), OGG1, PMS1, PMS2, polymerase delta1, polymerase epsilon, polymerase beta epsilon, polymerase eta, polymerase kappa, PTEN, RAD50, RAD51, RAD51C, RAD51D, SMAD4, STK11, TP53, XPA, XPC, XRCC2, XRCC3, XRCC4* | *BRCA1(9),BRCA2(11)* | Breast MRI, RRM and RRSO* |
|  |  |  | *TP53(2)* | Breast MRI, RRM* |
|  |  |  | *ATM(1)* | Breast MRI*, |
|  |  |  | *BRIP1(1), MSH2(1). RAD51C(1)* | RRSO* |
|  |  |  | *MUTYH(1)* | Pan colonoscopy¶ |
|  |  |  | *RAD50(2)* | Monitored for malignancy, particularly breast cancer in women and prostate cancer in men¶. |
|  |  |  | *FANCI(1)* | Regular blood counts and bone marrow aspirate/biopsy for morphology and others ¶ |

Breast MRI: breast magnetic resonance imaging; RRM: risk-reducing mastectomy; RRSO: risk-reducing salpingo-oophorectomy

* National Comprehensive Cancer Network. *NCCN Clinical Practice Guidelines in Oncology. Genetic/familial high-risk assessment: breast and*

*ovarian*. 2016; Available from: http://www.nccn.org/professionas/physician_gls/pdf/genetics_screening.pdf. P.28

## ¶GeneReviews^®^ [Internet].https://www.ncbi.nlm.nih.gov/books/NBK1176/ for *RAD50*; https://www.ncbi.nlm.nih.gov/books/NBK107219/ for *MUTYH*; https://www.ncbi.nlm.nih.gov/books/NBK1401/ for *FANCI*
